# Dynamics of Cough Frequency in Adults Undergoing Treatment for Pulmonary Tuberculosis

**DOI:** 10.1093/cid/cix039

**Published:** 2017-01-25

**Authors:** Alvaro Proaño, Marjory A. Bravard, José W. López, Gwenyth O. Lee, David Bui, Sumona Datta, Germán Comina, Mirko Zimic, Jorge Coronel, Luz Caviedes, José L. Cabrera, Antonio Salas, Eduardo Ticona, Nancy M. Vu, Daniela E. Kirwan, Maria-Cristina I. Loader, Jon S. Friedland, David A. J. Moore, Carlton A. Evans, Brian H. Tracey, Robert H. Gilman, for the Tuberculosis Working Group in Peru

**Affiliations:** 1 Escuela Profesional de Medicina, Facultad de Medicina Alberto Hurtado, and; 2 Laboratorio de Investigación en Enfermedades Infecciosas, Laboratorio de Investigación y Desarrollo, Facultad de Ciencias y Filosofía, Universidad Peruana Cayetano Heredia, and; 3 Asociación Benéfica PRISMA, Lima, Perú;; 4 Department of General Internal Medicine, Massachusetts General Hospital, Boston;; 5 Innovation For Health And Development, Laboratory of Research and Development, and; 6 Laboratorio de Bioinformática y Biología Molecular, Facultad de Ciencias y Filosofía, Universidad Peruana Cayetano Heredia, and; 7 Instituto Nacional de Salud del Niño San Borja, Lima, Perú;; 8 Department of Global Community Health and Behavioral Sciences, Tulane University, New Orleans, Louisiana,; 9 Division of Epidemiology and Biostatistics, Mel and Enid Zuckerman College of Public Health, University of Arizona, Tucson;; 10 Infectious Diseases and Immunity and Wellcome Trust Imperial College Centre for Global Health Research, Imperial College London, United Kingdom;; 11 Escuela Profesional de Ingeniería Física, Facultad de Ciencias, Universidad Nacional de Ingeniería,; 12 Servicio de Neumología, Hospital Nacional Alcides Carrión,; 13 Servicio de Neumología, Hospital Nacional Dos de Mayo,; 14 Facultad de Medicina, Universidad Nacional Mayor de San Marcos, and; 15 Servicio de Enfermedades Infecciosas y Tropicales, Hospital Nacional Dos de Mayo, Lima, Perú;; 16 Department of Internal Medicine, Cleveland Clinic, Ohio;; 17 Tuberculosis Centre, London School of Hygiene and Tropical Medicine, United Kingdom;; 18 Department of Electrical and Computer Engineering, Tufts University, Medford, Massachusetts, and; 19 Program in Global Disease Epidemiology and Control, Department of International Health, Bloomberg School of Public Health, Johns Hopkins University, Baltimore, Maryland

**Keywords:** tuberculosis, airborne transmission, infectiousness, cough, Peru.

## Abstract

**Background.:**

Cough is the major determinant of tuberculosis transmission. Despite this, there is a paucity of information regarding characteristics of cough frequency throughout the day and in response to tuberculosis therapy. Here we evaluate the circadian cycle of cough, cough frequency risk factors, and the impact of appropriate treatment on cough and bacillary load.

**Methods.:**

We prospectively evaluated human immunodeficiency virus–negative adults (n = 64) with a new diagnosis of culture-proven, drug-susceptible pulmonary tuberculosis immediately prior to treatment and repeatedly until treatment day 62. At each time point, participant cough was recorded (n = 670) and analyzed using the Cayetano Cough Monitor. Consecutive coughs at least 2 seconds apart were counted as separate cough episodes. Sputum samples (n = 426) were tested with microscopic-observation drug susceptibility broth culture, and in culture-positive samples (n = 252), the time to culture positivity was used to estimate bacillary load.

**Results.:**

The highest cough frequency occurred from 1 pm to 2 pm, and the lowest from 1 am to 2 am (2.4 vs 1.1 cough episodes/hour, respectively). Cough frequency was higher among participants who had higher sputum bacillary load (*P* < .01). Pretreatment median cough episodes/hour was 2.3 (interquartile range [IQR], 1.2–4.1), which at 14 treatment days decreased to 0.48 (IQR, 0.0–1.4) and at the end of the study decreased to 0.18 (IQR, 0.0–0.59) (both reductions *P* < .001). By 14 treatment days, the probability of culture conversion was 29% (95% confidence interval, 19%–41%).

**Conclusions.:**

Coughs were most frequent during daytime. Two weeks of appropriate treatment significantly reduced cough frequency and resulted in one-third of participants achieving culture conversion. Thus, treatment by 2 weeks considerably diminishes, but does not eliminate, the potential for airborne tuberculosis transmission.

## Summary

This is the first research to evaluate cough frequency continuously over 24-hour periods and to characterize associations with mycobacterial load and treatment. This study provides novel information on the circadian cycle of cough frequency and risk factors for increased cough frequency.

In 2015, there were an estimated 10.4 million new tuberculosis cases causing 1.4 million deaths worldwide [1]. The major means by which transmission occurs is believed to be through aerosolized *Mycobacterium tuberculosis* expelled from an infectious person. A series of classic experiments in the 1960s showed that the number of *M. tuberculosis* droplet nuclei formed by coughing greatly exceeded those formed by singing or speaking, and concluded that cough is the main pathway by which bacilli are transmitted from the lung into the environment [2].

Cough frequency has been suggested as a predictor of transmission risk, and high cough frequency late in treatment has been associated with treatment failure [3, 4]. However, a recent review highlighted the paucity of information related to the dynamics of cough in tuberculosis [3]. This review noted that the last study reporting the frequency of cough among patients undergoing tuberculosis treatment was conducted nearly 50 years ago and only included an 8-hour overnight assessment of 20 patients [5]. Though it is logistically convenient to monitor solely nocturnal cough patterns, it is not known whether daytime coughs show similar patterns.

In this prospective cohort study, we recorded cough frequency using an objective acoustic tool [6, 7], as is the current recommendation [8]. We surveyed participants before and during tuberculosis treatment to investigate (1) the circadian cycle, (2) risk factors associated with cough, and (3) the impact of appropriate treatment on cough frequency and mycobacterial burden.

## METHODS

### Study Design

The parent prospective cohort study followed adults (aged ≥18 years) with a clinically suspected diagnosis of pulmonary tuberculosis in 2 reference tertiary academic Peruvian Hospitals: Hospital Nacional Dos de Mayo and Hospital Nacional Daniel Alcides Carrión, for which a protocol detailing sample size, selection criteria, and detailed information on variables has been published [9]. Data for human immunodeficiency virus (HIV)–infected participants and those who did not have confirmed drug-susceptible pulmonary tuberculosis are being reported separately. For the current study, inclusion criteria were the subset of the parent study with sputum culture–positive tuberculosis confirmed to be susceptible to isoniazid and rifampicin (to reduce the risk of incorrect treatment confounding results) in participants confirmed to be HIV negative (due to the unknown effect of immunodeficiency on cough). The exclusion criterion was no adequate recording during the study period.

Clinicians treated patients for tuberculosis according to the Peruvian national guidelines, using direct observation of every treatment dose. Patient treatment was not modified by this study. They were followed in our study until 62 days after treatment initiation. Cough was recorded among all suspected tuberculosis cases at the time of participant enrollment. Participants were asked to complete a previously published questionnaire regarding their socioeconomic status [9].

This study was approved by the ethics committees of both participating hospitals, A.B. PRISMA and Universidad Peruana Cayetano Heredia (UPCH) in Lima, Peru; and Johns Hopkins University in Baltimore, Maryland.

### Cough Frequency Assessment

The Cayetano Cough Monitor (CayeCoM) device is a semiautomated ambulatory cough monitor that [9], along with our previously developed algorithm, identifies cough with a sensitivity of 75.5% and a Birring specificity [10] of 99.3% among adults with pulmonary tuberculosis [7]. All recordings that malfunctioned or were of poor sound quality due to high background noise were excluded. Recordings with a positive cough sound were further validated by 2 trained nurses who were responsible for listening to the portions of the recording identified by the algorithm to confirm each event as a “cough.” To reduce bias, recordings were randomly and blindly assigned to each nurse. For quality control purposes, a random subset of recordings was listened to by both nurses, and their agreement was assessed by calculating the κ statistic.

### Microbiological Assessment

Standard instructions were given to participants to collect early-morning sputum samples by deep coughing. We obtained a single morning sputum sample on the day that each participant started treatment (day 0), and on days 3, 7, 14, 21, 30, and 60 of treatment. All sputum samples underwent microbiological protocols at UPCH for auramine-stained smears, and the microscopic-observation drug susceptibility (MODS) broth culture assay incorporating drug susceptibility testing for isoniazid and rifampicin, as previously described [9, 11–13]. The numbers of acid-fast bacilli visualized by auramine microscopy were recorded as the smear grade. In MODS culture–positive samples, the number of days from inoculation to positive was recorded to assess viable bacillary load in sputum, and defined as time to positivity (TTP). TTP predicts treatment response and correlates with the number of colony-forming units (CFU) prior to and during treatment [14–16].

### Statistical Analysis

All analyses were conducted using Stata statistical software version 14 (StataCorp LP, College Station, Texas), under a 95% confidence level.

#### Cough Definitions

Cough was more likely to occur in clusters, termed salvos, rather than individually. Therefore, cough episodes were analyzed rather than individual cough events. A cough episode was defined as all consecutive cough events that occurred without a cough-free pause of 2 seconds or more [7]. Cough frequency was defined as the number of cough episodes/hour.

Based on previous findings by other groups [17, 18], which used cough events rather than episodes, we defined “no cough” as ≤0.7 cough events/hour, and cough cessation was defined as the first of 2 consecutive recordings with no cough.

#### Mycobacterial Load Calculations

To estimate CFUs from TTP, we used the equation [log_10_ CFU = 5.1 − (0.16×TTP)], based on our group’s data on quantitative cultures [15, 19]. Therefore, CFUs were estimated only for positive cultures, and all negative and indeterminate cultures were excluded from CFU analyses. Smear conversion was defined as the first negative smear with no subsequent positive smear; culture conversion was defined as the first negative culture with no subsequent positive culture.

##### Circadian cycle of cough frequency.

Cough frequency was modeled using nested negative binomial regression with random effects with an exchangeable correlation structure at the level of participant and treatment day, and a robust variance estimate. This was chosen based on a comparison of quasi-likelihood under the independence model criterion statistics between models with alternative correlation structures. To describe circadian cycles of cough, this model was adjusted for the hour of the day using harmonic sine and cosine terms as shown in Supplementary Equation 1. To test whether the circadian cycle varied with duration of treatment, models were fitted (1) separately by treatment day and (2) adjusting for treatment day, with interactions between treatment day and sine/cosine terms.

##### Risk factors associated with increased cough frequency.

Random-effects negative binomial regression with a participant-level random intercept was used to evaluate the association between cough frequency and mycobacterial load, as well as prior participant-reported tuberculosis, and socioeconomic status (monthly income). A final multivariable model was created, adjusting for risk factors found to be significant (*P* ≤ .05) in univariable analysis. In addition, because the relationship between the duration of treatment and cough frequency was nonlinear, both day of treatment and day of treatment squared were included as independent variables. TTP provides a more precise quantification of bacillary load, so it was preferred over smear- or culture-positive status in the multivariable analysis.

##### Impact of appropriate treatment.

The effects of the duration of appropriate treatment on cough frequency, smear grade, and MODS culture conversion were analyzed using Cox proportional hazard models. In this model, cough frequency was assessed as (1) a 2-fold reduction compared to the pretreatment cough frequency, as previously used by Loudon and Spohn [5], and (2) as no cough.

#### Feasibility of Shorter Recordings

Cough frequency calculated over a full day (≥23.5 hours) was compared to cough frequencies calculated over shorter periods (2- to 12-hour periods during the day). Cough recordings were split randomly into a discovery set (70% of recordings) and a validation set (30% of recordings). Using the discovery dataset, cough frequency was calculated during 2- to 12-hour periods throughout the day, and Spearman (nonparametric) correlation between total cough episodes occurring over each shortened window and total cough episodes over the full ≥23.5 hours was calculated. The time of day that correlated most highly with 24-hour cough was identified. This result, and the individual intraclass correlation coefficient, was then tested in the validation dataset.

## RESULTS

### Demographics and Pretreatment Assessment of Microbiology

**Figure 1.**
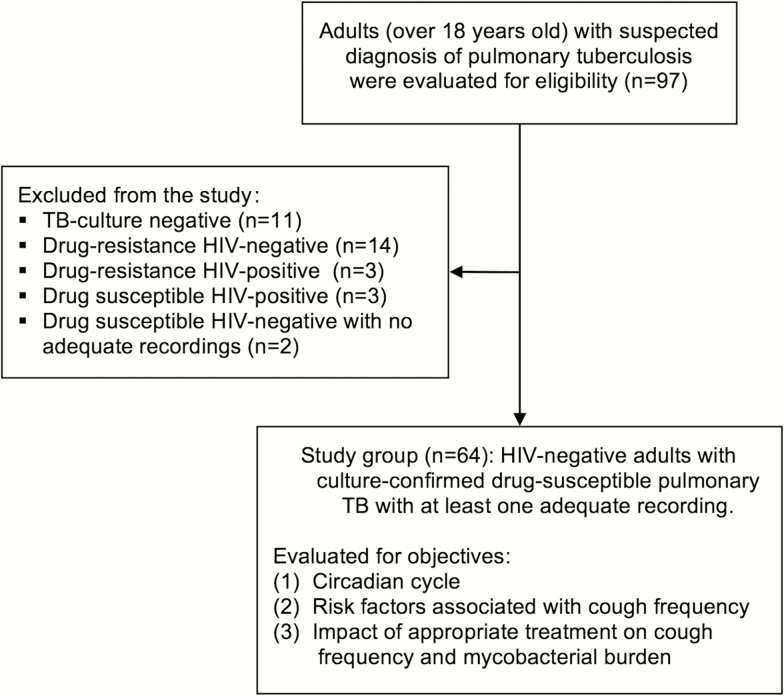
Flowchart for the Cayetano Cough Monitor Study. Abbreviations: HIV, human immunodeficiency virus; TB, tuberculosis.

Ninety-seven adults were enrolled in the parent study, who contributed with 957 recordings, with 685 of 1642 (42%) recordings excluded for technical reasons (Supplementary Table 1). Of these, 66 met inclusion criteria for the current study, and 2 were excluded because they had no adequate cough recording, so the study group consisted of 64 participants (Figure 1). Of these participants, all had at least 1 positive MODS culture, which included their first sputum sample for 95% of participants. Baseline demographic data are shown in Table 1.

**Table 1.** Baseline Demographic Characteristics of Study Participants

Variable	Study Group
Total participants	64
Male participants (%, 95% CI)	44 (69%, 57%–80%)
Median age, y, at study enrollment (IQR)	32 (22–44)
Pretreatment culture positive (%, 95% CI)	61 (95%, 90%–100%)
Pretreatment culture negative (%, 95% CI)	1.0 (1.6%, 0.0–4.7%)
Pretreatment indeterminate culture (%, 95 CI)	2.0 (3.1%, 0.0–7.5%)
Median pretreatment TTP, d (IQR)	7.0 (6.0–9.0)
Pretreatment negative smear (%, 95% CI)	19 (30%, 18%–41%)
Pretreatment paucibacillary smear (%, 95% CI)	5.0 (7.8%, 1.1%–15%)
Pretreatment smear + (%, 95% CI)	19 (30%, 18%–41%)
Pretreatment smear ++ (%, 95% CI)	8.0 (13%, 4.2%–21%)
Pretreatment smear +++ (%, 95% CI)	13 (20%, 10%–30%)
Drug-susceptible participants (%, 95% CI)	64 (100%, 100%–100%)
Total hours of recording	12108
Total participant-days of recordings^a^	661 (670 unique recordings)
Total complete daily recordings^b^	267 (267 unique recordings)

Patient characteristics and microbiological data corresponding to study group.

Abbreviations: +, 20–199 acid-fast bacilli per 40 fields at 400× magnification; ++, 5–50 acid-fast bacilli per field at 400× magnification; +++, >50 acid-fast bacilli per field at 400× magnification; CI, confidence interval; IQR, interquartile range; TTP, time to positivity of microscopic-observation drug susceptibility culture.

^a^Total participant-days of recordings is the number of days within the study that contributed with recordings; if a participant had multiple unique recordings on the same day, it will still contribute to only 1 participant-day of recording.

^b^Total complete daily recordings were recordings that were at least 23.5 hours long.

### Cough Validation and Characteristics

The median length of cough episodes was 0.61 seconds (interquartile range [IQR], 0.26–2.2), and 90% were <3.9 seconds long. Fifty percent of episodes contained only a single cough event, 24% had 2 cough events, and the remaining 26% contained ≥3 events. The maximum number of total cough events in a single episode was 21.

There was good agreement among the 43% subset of recordings reviewed by both nurses, with a Cohen κ statistic of 0.93.

#### Circadian Cycle of Cough Frequency

**Figure 2.**
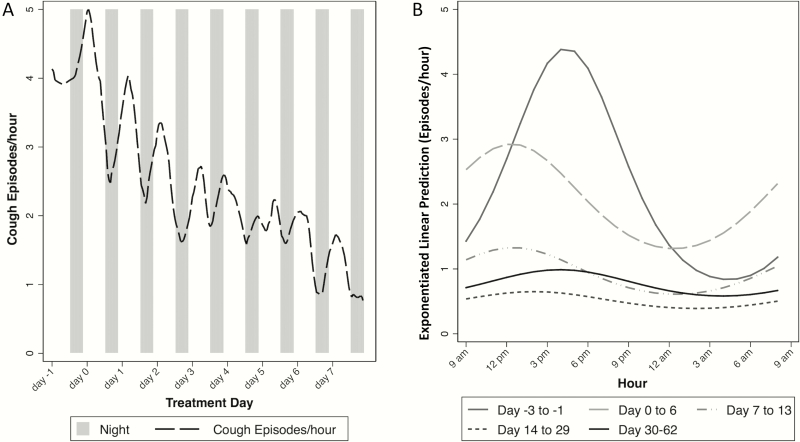
Circadian cycle of cough frequency during treatment for study group. *A*, Smoothed trends in cough from day –1 to day 7 of treatment. Each day begins at 9 am, as this is the time when recordings began. *B*, Separate negative binomial generalized estimating equation models fitted for each day following treatment. All recordings, regardless of total length, were included (n = 12108 hours of recording). Random-effects modeling was used to adjust for study participant. Circadian cycles of cough were reflected by sine/cosine terms.

Based on model estimates, the highest pretreatment cough frequency occurred from 1 pm to 2 pm and the lowest from 1 am to 2 am (2.4 vs 1.1 cough episodes/hour, respectively). Thus, cough episodes were twice as frequent during daytime as nighttime. This circadian cycle was present throughout the study period (Figure 2). For example, after 14 days of treatment, cough episodes per hour were 1.5 from 1 to 2 pm vs 0.73 from 1 to 2 am.

#### Risk Factors Associated With Increased Cough Frequency

Cough frequency was independently associated with day of treatment (rate ratio [RR] per 10 days, 0.37; *P* < .01), day of treatment squared (RR, 1.3; *P* < .001), and TTP (RR, 0.93; *P* < .01) in the multivariable model (Table 2). Pretreatment cough frequency was correlated with cough frequency on treatment day 3 (Spearman ρ, 0.47; 95% confidence interval [CI], .095–.73; *P* = .02); but was not statistically significantly correlated with cough frequency at later time points. Cough frequency decreased with increasing duration of treatment (Supplementary Figure 1). Cough frequency was not independently significantly associated with income or prior tuberculosis (Table 2).

**Table 2.** Risk Factors for Cough Frequency in Univariable and Multivariable Negative Binomial Model Adjusting for Study Participant

Risk Factor	Univariable Analysis (64 Participants, 661 Observations)			Multivariable Analysis (59 Participants, 173 Observations)		
	RR	*P* Value	95% CI	RR	*P* Value	95% CI
Treatment day (per 10 d)	0.68	<.001	.62–.75	0.37	.001	.20–.68
Treatment day squared	0.97	<.001	.95–.98	1.28	<.001	1.11–1.46
Monthly income (Peruvian soles)	1.00	.942	1.00–1.00			
Prior tuberculosis, yes/no	1.02	.719	.93–1.11			
MODS culture positive	2.34	<.001	1.70–3.24			
Time to positivity, d	0.90	<.001	.87–.94	0.93	.005	.89–.98
Time to positivity (categorical)						
5–7 d	Reference					
8–10 d	0.75	.115	.52–1.07			
≥11 d	0.47	<.001	.33–.68			
Smear (categorical)						
Negative	Reference					
Paucibacillary	1.62	.052	1.00–2.64			
+	2.65	<.001	1.89–3.71			
++	3.78	<.001	2.58–5.54			
+++	3.89	<.001	2.55–5.94			
Sex, female	1.33	.032	1.02–1.74	1.23	.292	.83–1.83
Age, y (per 10 y)	1.41	<.001	1.29–1.54	1.11	.100	.98–1.25

Results of the univariable and multivariable negative binomial models examining cough frequency. A random-effects negative binomial model was used to adjust for study participant.

Abbreviations: +, 20–199 acid-fast bacilli per 40 fields at 400× magnification; ++, 5–50 acid-fast bacilli per field at 400× magnification; +++, >50 acid-fast bacilli per field at 400× magnification; CI, confidence interval; MODS, microscopic-observation drug susceptibility assay; RR, rate ratio.

#### Impact of Appropriate Treatment

**Figure 3.**
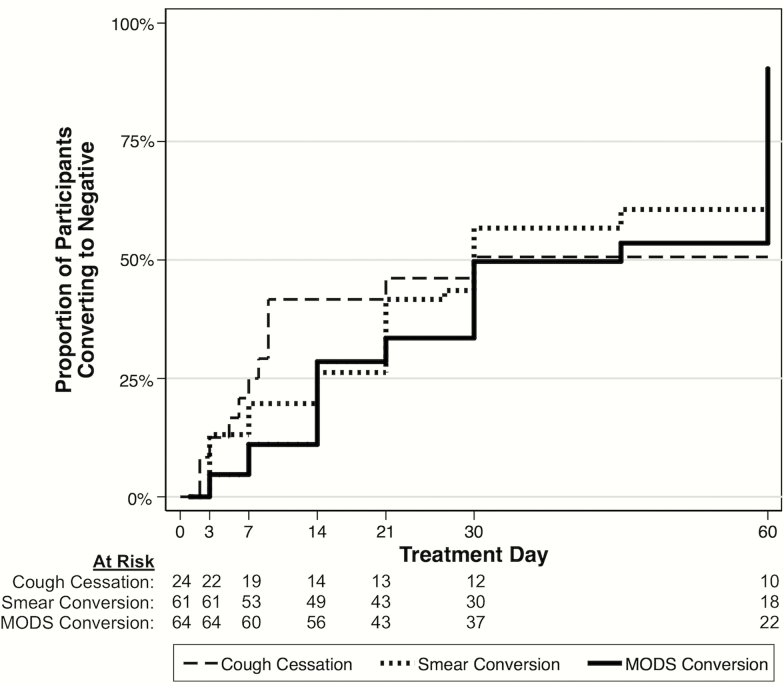
Kaplan-Meier curves for time to coughing cessation and microbiological conversion in study group. Cough cessation represents the time to the first of 2 consecutive recordings with a cough frequency of ≤0.7 cough events per hour (considered “no cough”); by day 14, the probability of cough cessation was 42% (95% confidence interval [CI], 25%–64%), and by day 60 the probability was 51% (95% CI, 33%–72%). Smear conversion represents the time to the first negative smear with no subsequent positive smear; by day 14, the probability of smear conversion was 26% (95% CI, 17%–39%), and by day 60 the probability was 85% (95% CI, 73%–93%). Microscopic-observation drug susceptibility (MODS) culture conversion represents time to the first negative culture with no subsequent positive culture; by day 14, the probability of MODS culture conversion was 29% (95% CI, 19%–41%), and by day 60 the probability was 94% (95% CI, 85%–98%).

Pretreatment median cough episodes per hour was 2.3 (IQR, 1.2–4.1), which after 14 days of treatment decreased to 0.48 (IQR, 0.0–1.4; *P* < .001) and by the end of the study decreased to 0.18 (IQR, 0.0–0.59; *P* < .001 compared with pretreatment). By 2 weeks of continuous treatment, the probability of cough cessation, smear conversion, and MODS culture conversion was 42% (95% CI, 25%–64%), 26% (95% CI, 17%–39%), and 29% (95% CI, 19%–41%) respectively; by the end of the study the probabilities increased to 51% (95% CI, 33%–72%), 85% (95% CI, 73%–93%), and 94% (95% CI, 85%–98%), respectively (Figure 3).

The median time to halving of cough frequency was 7.0 days, and cough frequency had at least halved for 73% of participants by day 14. Participants with no cough pretreatment continued to have no cough throughout treatment. A higher pretreatment cough frequency was associated with a faster halving of cough frequency during treatment (hazard ratio, 1.2; *P* = .02). Other pretreatment factors (eg, age, sex) were not significantly associated with time to halving of cough frequency or time to achieve no cough. When cough trends were examined on a participant-by-participant basis, most showed a strong and immediate decrease in cough frequency after treatment, following the overall trend.

There was no statistically significant association between pretreatment cough frequency (median, 2.3 cough episodes/hour; IQR, 1.2–4.1) and time to smear conversion (median, 21 days of treatment; IQR, 6–32) or culture conversion (median, 29 days; IQR, 13–61). Throughout the study, the cough frequency on days when participants had positive MODS cultures was approximately double the cough frequency on days when cultures were negative (univariate analysis RR, 2.3; *P* < .001). When assessing bacillary load, TTP was inversely associated with cough frequency such that as TTP increased indicating reduced bacillary load, cough frequency decreased (RR, 0.93; *P* < .01) (Table 2). The relationship between cough frequency, TTP, and estimated log_10_ CFU over time is shown in Supplementary Figure 2. There was a significant association (*P* < .001) between TTP and sputum smear positivity in pretreatment samples (Supplementary Figure 3).

Among the 41% (26/64) of participants with matched cough recordings and sputum samples available pretreatment, median cough frequency was 2.3 cough episodes per hour (IQR, 1.2–4.1); median TTP was 6.0 days (IQR, 6.0–7.0), and 12% initially had no cough (Supplementary Table 2). At 2 weeks of treatment, 77% (49/64) of participants had matched recordings and sputum samples, with a median cough frequency of 0.43 (IQR, 0.0–1.1) and a median TTP of 11 (IQR, 10–13). At day 60, 65% (15/23) of participants with matched samples had no cough.

Comparing participants who were lost to follow-up on or before day 14 to those who continued past this point, there were no statistically significant differences in baseline cough (2.2 vs 2.4 cough episodes/hour; *P* = 1) initial smear results (++: 14% vs 12%; *P* = .16); or baseline TTP (6.5 vs 7.0; *P* = .11). Those who were lost to follow-up were also similar in sex, were of similar age, and were no more likely to have had prior tuberculosis.

### Feasibility of Shorter Recordings

Among 4-hour recordings, afternoon recordings (2–6 pm) had the highest correlation with 24-hour recordings (ρ = 0.86; standard error = 0.04). The least representative 4-hour period occurred in the evening (10 pm–2 am). Daytime shorter recordings correlated best with 24-hour recordings (Supplementary Table 3).

## DISCUSSION

Tuberculosis transmission occurs by aerosol spread, and the bacterial burden within sputum is often used as a proxy for infectiousness [20]. However, airborne tuberculosis transmission can only occur if there is a mechanism for distribution, such as expulsion through cough. Cough frequency in tuberculosis has been poorly studied with only a single study reported nearly 50 years ago. This study only observed patients at night and did not evaluate subject-specific dynamics over time [5]. To ensure adequate prevention strategies, an improved understanding of cough dynamics, before and during treatment, is required.

Our group used a previously validated cough monitor and algorithm to record cough episodes from 64 HIV-negative participants diagnosed with drug-susceptible pulmonary tuberculosis. We observed that cough frequency varied throughout the day, with the highest frequency in the afternoon, a time of day when patients are likely to be active outside their homes, and lowest at nighttime, likely during sleep [21]. When comparing our results to those of Loudon and Spohn [5], we found a similar pattern of decrease in nighttime 8-hour cough frequency (11 pm–7 am) over our study period. We also found that shorter periods of cough recording have reasonable agreement with 24-hour recordings [22]. Our study also shows that cough, at a lower frequency, can continue within 2 months of treatment, supporting previous results [4, 23]. However, it should be noted that cough alone can be a nonspecific symptom for tuberculosis; thus, both cough frequency and sputum MODS cultures were assessed.

Increased cough frequency was associated with MODS culture positivity, as well as decreased time to positivity, a surrogate for bacterial load [15, 16]. This suggests that cessation of cough is associated with sputum bacillary load and MODS culture conversion to negative during the first 2 months of treatment, which suggests that cough reflects treatment response [4]. Current guidelines note that following 2 weeks of tuberculosis treatment, infectiousness is greatly reduced [24–26], despite the presence of viable pathogens far beyond this time [27]. This implies that infectivity not only depends on microbiology positivity, but also on other factors such as cough frequency [28]. In support of this, we found that cough frequency dropped rapidly in the first days of treatment. Estimated CFU counts dropped faster within the first days of treatment [29–31], alongside an exponential decline in cough frequency. This supports the observation that within the first days of treatment a large proportion of the actively growing mycobacteria are killed [29], and that effective treatment may rapidly diminish transmission [32].

Of the 26 participants who provided concurrent cough recordings and sputum samples prior to commencing treatment, almost one-eighth had no cough. “No cough” patients with pulmonary tuberculosis have been described previously [33, 34]; however, this is the first time this has been quantified. The World Health Organization, the International Union Against Tuberculosis and Lung Disease, and the Royal Netherlands Tuberculosis Association define case detection when cough lasts between 2 and 3 weeks, the entry point for routine tuberculosis diagnostic screening [35]. Thus, restriction of tuberculosis diagnostic testing to those defined within the current “case detection” definition worldwide may miss a substantial number of patients with active pulmonary disease, and therefore screening must also consider that cough might not be present. By not relying solely on 2–3 weeks of cough as the entry point for screening, this will increase the number of diagnosed and treated patients, which will increase the positive impact of the tuberculosis program, albeit at the cost of more people being eligible for screening.

A strength of this study is that it used an objective cough monitor that has been validated in adults with tuberculosis [6, 7], with a sensitivity of 75.5%, comparable to that of other semiautomated methods [36, 37], and utilized day-long recordings that enabled determination of cough frequency by hour. A limitation of our method is the large proportion of recordings that could not be processed due to relatively high levels of background noise, and the relatively smaller number of early-morning (6–9 am) recordings available. We are working to solve this technical issue by the development of a second-generation accelerometer-based cough monitor [38]. In addition, we did not quantify CFUs but instead mathematically estimated CFUs from other quantitative data in MODS cultures [15, 19]. Our formula provides similar results to those obtained by another group who modeled CFUs from TTP in Mycobacteria Growth Indicator Tube (MGIT) culture [16]. Another limitation is that participants enrolled in this study were recruited from 2 tertiary academic hospitals, and might not reflect the broader population of tuberculosis in the community.

The current convention for infection control is that, following 2 weeks of adequate treatment, patients with tuberculosis pose a significantly reduced risk of onward transmission, so it is safe to consider discontinuing infection control practices including respiratory isolation [24–26, 39]. Despite the fact that bacterial growth can occur from sputum obtained as late as 60 days into adequate treatment [27], our data show a rapid drop in cough frequency, which is associated with microbiological conversion. This supports earlier findings which show that pulmonary tuberculosis transmission is greatly reduced once adequate treatment starts [32, 39, 40], and suggests that tuberculosis treatment response could be indirectly measured by assessing cough frequency.

## Supplementary Data

Supplementary materials are available at *Clinical Infectious Diseases* online. Consisting of data provided by the authors to benefit the reader, the posted materials are not copyedited and are the sole responsibility of the authors, so questions or comments should be addressed to the corresponding author.

## Data sharing statement

Data from this study is publicly available through the Dryad Digital Repository at http://dx.doi.org/10.5061/dryad.gv234

## Supplementary Material

supplementary_03_01_17

## Notes

***Author contributions.*** All authors were involved in the study design and drafting the manuscript for intellectual content, and all reviewed the final manuscript before submission. M. A. B. and J. W. L. directly contributed to the study design and were responsible for supervision of data gathering. A. P., G. O. L., D. B., and M. Z. directly contributed to data management and statistical analysis. The corresponding authors had full access to all the data in the study and had final responsibility for the decision to submit for publication.

***Acknowledgments.*** This work is dedicated to the memory of Luz Caviedes, who passed away in November 2012. We thank the participants of this study for their time and collaboration. We also thank Donald Corle (National Institutes of Health [NIH]) who helped with initial sample size calculations.

***Disclaimer.*** The funding sources had no role in the writing of the manuscript or the decision to submit it for publication. No payments were made for writing the article.

***Financial support.*** This work was supported by the NIH (5D43TW006581, 5R21AI094143-02, and 5D43TW009349-03) and Grand Challenges Canada (0539-01-10). Contributions by coauthors were funded as follows: Wellcome Trust (award numbers 078067/Z/05/Z to D. A. J. M., 105788/Z/14/Z and 201251/Z/16/Z to S. D. and C. A. E.); Imperial College Biomedical Research Centre to S. D., J. S. F., and C. A. E.; Joint Global Health Trials (award number MR/K007467/1 to S. D., C. A. E., and R. H. G.); Bill & Melinda Gates Foundation (OPP1118545 to S. D. and C. A. E.); Innovation for Health And Development funding (to C.A.E.).

***Potential conflicts of interest.*** All authors: No potential conflicts. All authors have submitted the ICMJE Form for Disclosure of Potential Conflicts of Interest. Conflicts that the editors consider relevant to the content of the manuscript have been disclosed.

## APPENDIX

Other members of the Tuberculosis Working Group in Peru include:

Lilia Cabrera and Marco Varela (Asociación Benéfica PRISMA, Lima, Peru); María Prado and Richard Rodríguez (Hospital María Auxiliadora, Lima, Peru); Aldo Vivar (Hospital Nacional Arzobispo Loayza, Lima, Peru); Jesus Chacaltana (Hospital Nacional Daniel Alcides Carrion, Lima, Peru); Felix Llanos and Marco Ñavincopa (Hospital Nacional Dos De Mayo, Lima, Peru); Eduardo Sanchez (Hospital Nacional Hipólito Unanue, Lima, Peru); Louis Grandjean and Roderick Escombe (Imperial College London, United Kingdom); José Gómez-Márquez (Massachusetts Institute of Technology, Cambridge); Gustavo Hernández-Córdova and Richard Oberhelman (Tulane University, New Orleans, Louisiana); Patricia Fuentes and Patricia Sheen (Universidad Peruano Cayetano Heredia, Lima, Peru); and nurses from the Peruvian National Tuberculosis Program.
