# Supplementary material for: Dynamics of Cough Frequency in Adults Undergoing Treatment for Pulmonary Tuberculosis
Source: Clin Infect Dis. 2017 Jan 25;64(9):1174–81. doi: 10.1093/cid/cix039 (PMC5399950; doi:10.1093/cid/cix039)
Supplement: supplementary_03_01_17 [file cix039_suppl_supplementary_03_01_17.pdf]

Supplementary Equation 1:

$$\log(cough\ episodes/hour) = b_j + \beta_0 + \beta_1 sine\left(\frac{2\pi t}{24}\right) + \beta_2 cosine\left(\frac{2\pi t}{24}\right) + \beta_3 treatment\ day + \varepsilon_{ij}$$

In the equation above, j represents the participant, i represents the day of the recording, and t is the hour of the day.

Supplementary Table 1: Recordings in All Enrolled Participants and in Study Group

	**All Enrolled**	**Study Group**
**Number of participants evaluated**	97	66
**Total recordings**	1642	1122
**Recordings excluded from analysis (%)**	685 (42%)	452 (40%)
… recording malfunction	45	36
… recordings with high background noise	485	308
… MP3 to WAV conversion error	18	14
… recordings not checked by nurse	58	38
… recordings started after 24 hours	16	10
… recordings shorter than 1 hour	63	46
**Participants with adequate recordings**	94	64
**Recordings contributing to analysis**	957 (936 total days)	670 (661 total days)
**Hours contributing to analysis**	17,412	12,108

From the 97 enrolled participants in the parent study, there were 3 participants who did not have a single adequate recording during the study period due to high background noise. The 3 excluded comprised of 2 participants (recordings=4) who were drug-susceptible HIV-negative and 1 participant (recordings=4) who was TB-culture negative. Therefore, the number of participants with adequate recordings in all enrolled participants was 97% (94/97), and for the HIV-negative with culture-confirmed drug-susceptible pulmonary TB study group was also 97% (64/66).

Supplementary Table 2: Cough, Culture, and Smear in Participants with Matched Samples in Study Group

**Day of Treatment**	**Pre-tx**	**3**	**7**	**14**	**21**	**30**	**60**
**Number of participants evaluated**	26	57	52	49	32	30	23
**Participants with "no cough" (%)**	12%	40%	37%	49%	66%	57%	65%
**Participants with culture-positive samples (%)**	96%	86%	7.7%	53%	47%	43%	0.0%
**Participants with culture-negative samples (%)**	0.0%	3.5%	9.6%	37%	28%	50%	96%
**Participants with indeterminate culture samples (%)**	3.9%	11%	83%	10%	25%	6.7%	4.4%
**Participants with smear negative samples (%)**	27%	23%	37%	47%	56%	60%	87%
**Participants with smear paucibacillary samples (%)**	7.7%	7.0%	5.8%	12%	9.4%	10%	4.4%
**Participants with smear + samples (%)**	31%	21%	21%	25%	22%	20%	8.7%
**Participants with smear ++ samples (%)**	3.9%	23%	23%	12%	13%	10%	0.0%
**Participants with smear +++ samples (%)**	31%	26%	13%	4.0%	0.0%	0.0%	0.0%
**Median cough frequency in participants**	2.3	0.83	0.66	0.43	0.088	0.24	0.18
**Median cough frequency in culture-positive participants**	2.4	1.0	0.67	0.96	0.53	0.47	-*
**Cough >0.7 in culture-positive participants (%)**	92%	63%	65%	65%	53%	62%	-*
**Median TTP in participants**	6.0	10	11	11	12	14	-*
**Median TTP in participants with "no cough"**	5.5	9.5	12	13	14	17	-*
**Median TTP in participants with cough >0.7**	6.0	10	11	10	11	12	-*
**Median $\log_{10}$CFU in participants**	4.1	3.5	3.3	3.3	3.2	2.9	-*
**Median $\log_{10}$CFU in participants with "no cough"**	4.2	3.6	3.2	3.0	2.9	2.4	-*
**Median $\log_{10}$CFU in participants with cough >0.7**	4.1	3.5	3.3	3.5	3.4	3.2	-*

Only participants with both cough and sputum data on a given day were included. "No cough" was defined as ≤0.7 cough events per hour. Cough frequency is based on cough episodes per hour. Estimated-CFU counts over time, based on our equation $\log_{10}\text{CFU} = 5.1 - (0.16\times\text{TTP})$, so only included positive cultures. No culture positive samples were found at day 60. -* represents no observations. All values rounded to two significant figures.

Supplementary Table 3: Correlation Between Short Recordings and 24-Hour Recordings in Study Group

		Discovery Set (n = 178)		Validation Set (n = 89)	
**Time Interval**	**Observation**	**Window**	**Spearman's ρ (se)**	**Spearman's ρ (se)**	**ICC**
2 hours	Best	2 p.m. – 4 p.m.	0.79 (0.05)	0.73 (0.07)	0.60
2 hours	Worst	11 p.m. - 1 a.m.	0.44 (0.07)	0.35 (0.10)	0.14
4 hours	Best	2 p.m. - 6 p.m.	0.86 (0.04)	0.86 (0.06)	0.67
4 hours	Afternoon	12 p.m. - 4 p.m.	0.84 (0.04)	0.85 (0.06)	0.75
4 hours	Morning	9 a.m. - 1 p.m.	0.77 (0.05)	0.83 (0.06)	0.66
4 hours	Worst	10 p.m. - 2 a.m.	0.54 (0.06)	0.41 (0.10)	0.27
8 hours	Best	12 p.m. - 8 p.m.	0.92 (0.03)	0.93 (0.04)	0.84
8 hours	Working*	9 a.m. - 5 p.m.	0.89 (0.04)	0.91 (0.04)	0.84
8 hours	Worst	10 p.m. - 6 a.m.	0.76 (0.05)	0.67 (0.08)	0.79
12 hours	Best	11 a.m. - 11 p.m.	0.95 (0.02)	0.93 (0.04)	0.90
12 hours	Worst	6 p.m. - 6 a.m.	0.88 (0.04)	0.83 (0.06)	0.86

Correlation (Spearman's rho and standard error) and intra-class correlation coefficient (ICC) between cough as recorded over 2-12 hour windows, versus 24-hour cough. All cough recordings that were complete to within 23.5 hours were included. Working* represents standard working hours (9 a.m. – 5 p.m.).

Supplementary Figure 1: Daytime and Nighttime Cough Frequency in Study Group

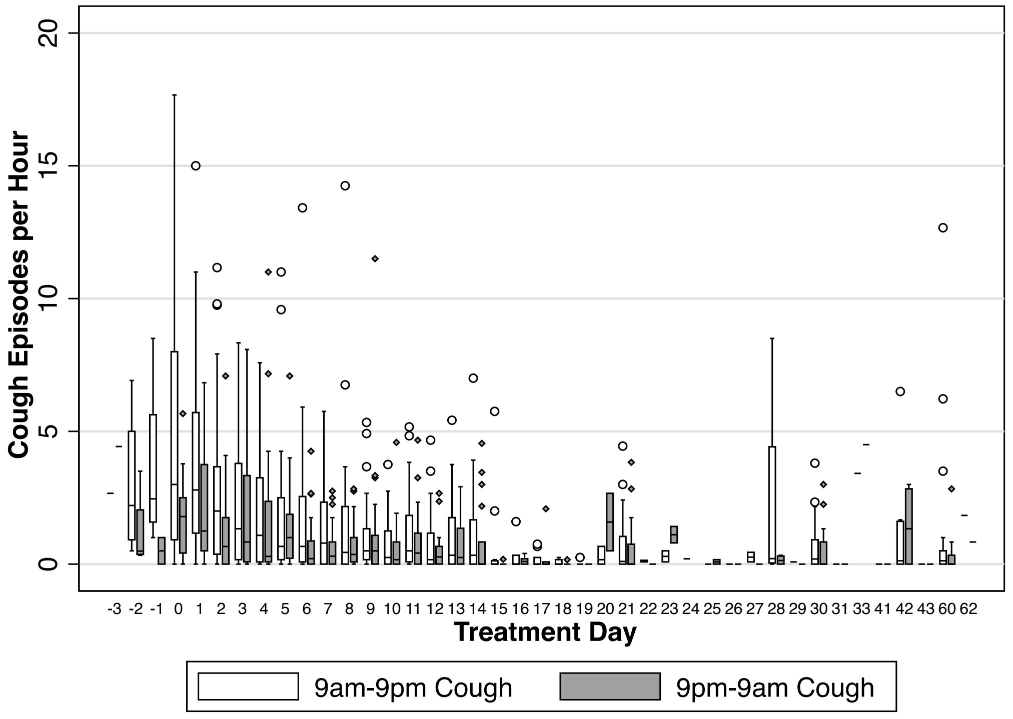

Supplemental Figure 1 shows daytime and nighttime cough frequency based on 12-hour intervals (9 a.m. – 9 p.m. and 9 p.m. – 9 a.m.); from 3 days prior to treatment until treatment day 62 (study period).

Supplementary Figure 2: Cough Frequency, Time to Positivity and Estimated-CFU by Days of Treatment in Study Group

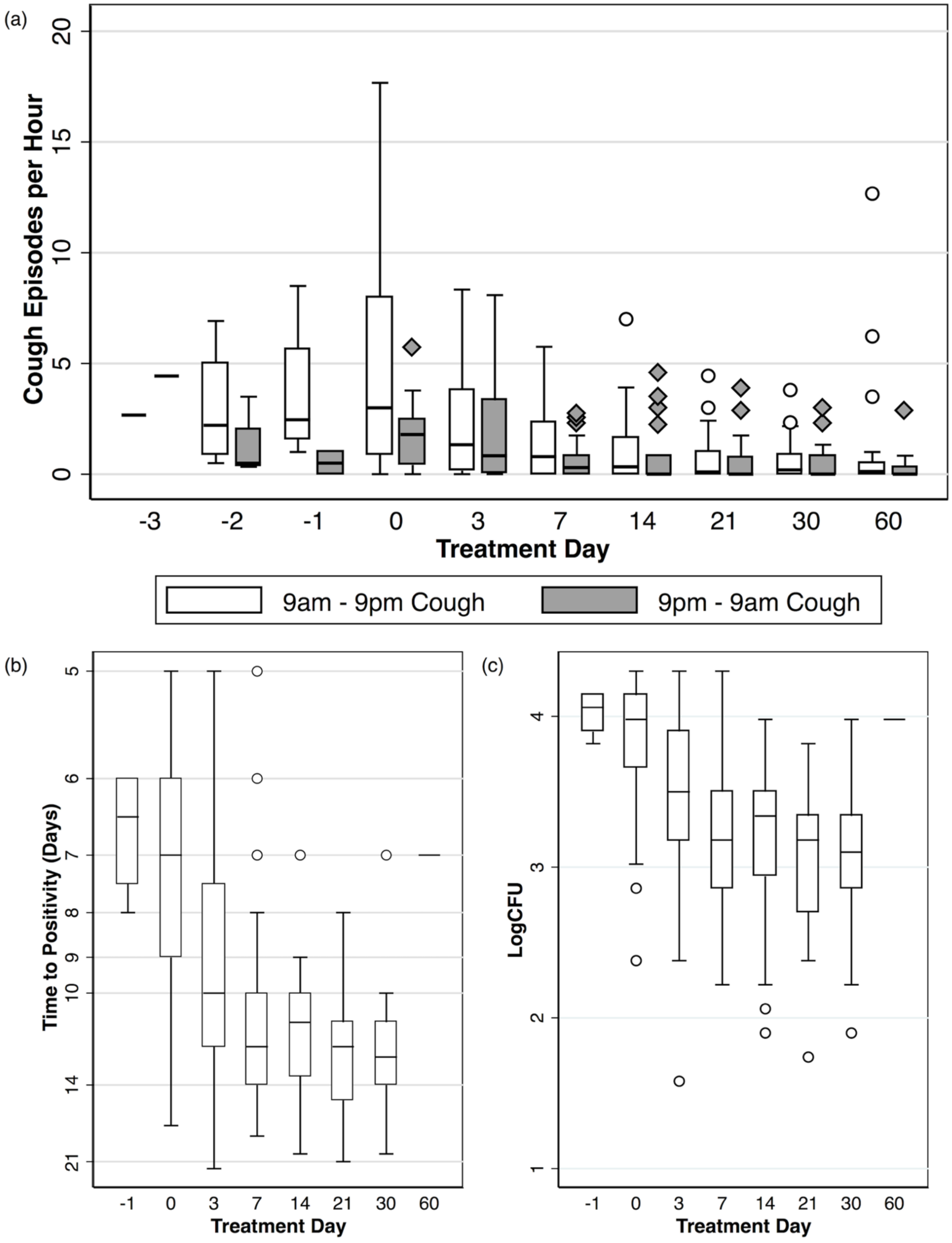

Supplementary Figure 2(a) shows daytime and nighttime cough frequency based on 12-hour intervals (9 a.m. – 9 p.m. and 9 p.m.–9 a.m.); this is only graphed for days on which sputum samples were collected.
Supplementary Figure 2(b) shows changes in time to positivity (TTP) at these same time points: the actual days to positivity are presented in an inverse scale (1/TTP). This represents longer time to positivity as treatment continues.
Supplementary Figure 2(c) shows estimated-CFU counts over time, based on our equation $\log_{10}\text{CFU} = 5.1 - (0.16 \times \text{TTP})$, so only included positive cultures.

Supplementary Figure 3: Box Plot of Time to Positivity and Estimated-CFU by Smear Results in Pre-Treatment Samples from Study Group

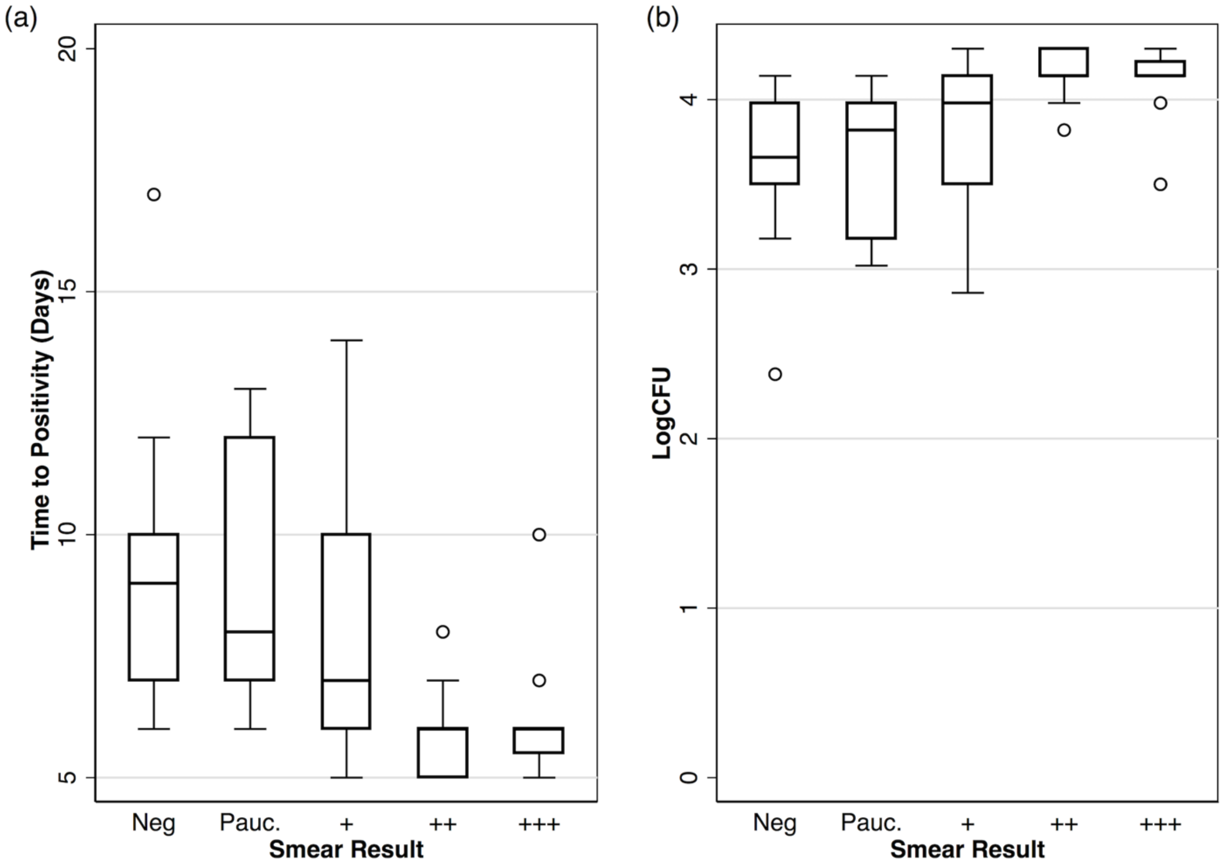

Supplementary Figure 3(a) shows a boxplot between time to positivity (TTP) by smear results in all culture-positive pre-treatment sputum samples. The correlation between TTP and smear had a Spearman rho of -0.53, and a $p<0.001$. **Supplementary Figure 3(b)** shows a boxplot between estimated-CFU counts by smear results in all culture-positive pre-treatment sputum samples. Estimated-CFU counts were obtained by the equation $\log_{10}CFU = 5.1 - (0.16\times TTP)$. The correlation between estimated-CFU and smear had a Spearman rho of 0.53, and $p<0.001$.
